# Exploring engagement patterns in a reproductive health app using the Technology Acceptance Model

**DOI:** 10.3389/frph.2026.1904798

**Published:** 2026-08-17

**Authors:** Alissa Kazakoff, Patricia K. Doyle-Baker

**Affiliations:** 1 Human Performance Lab, Faculty of Kinesiology, University of Calgary, Calgary, AB, Canada; 2 Alberta Children’s Hospital Research Institute, University of Calgary, Calgary, AB, Canada; 3 O’Brien Institute of Public Health, University of Calgary, Calgary, AB, Canada

**Keywords:** menstrual cycle tracking, menstrual literacy, mHealth, perceived ease of use, Technology Acceptance Model, user engagement

## Abstract

**Background:**

Menstrual tracking apps are widely used, yet research on what drives sustained engagement remains limited. Most studies rely on self-reported use, with few incorporating real-time behavioural data or validated theoretical frameworks such as the Technology Acceptance Model (TAM).

**Objective:**

This study examined associations between engagement with a reproductive health app offering menstrual tracking functionality and TAM constructs over a 90-day period using both survey and direct app usage data. Changes in menstrual literacy were also assessed to evaluate the app's educational impact.

**Methods:**

Participants (n = 293) were recruited via social media and completed baseline and follow-up surveys including TAM measures of perceived usefulness (PU), perceived ease of use (PEOU), trust, and menstrual literacy indicators (confidence and information sufficiency). Real-time app engagement data were collected continuously, and participants were categorized into high- and low-engaged groups based on actual use. Paired t-tests and multivariate regression analyses were used to assess changes over time and differences between groups.

**Results:**

At baseline, no differences were observed in TAM constructs between high- and low-engaged users. By follow-up, PEOU declined significantly in low-engaged users while high-engaged users maintained higher perceived ease of use (p < 0.05). Confidence in menstrual knowledge increased in both groups whereas information sufficiency did not change significantly. Feature usage showed that 98% tracked menstrual cycles, 73% tracked symptoms, and 42% tracked exercise. The app was rated as moderate quality (uMARS mean=3.1).

**Conclusion:**

Integrating TAM constructs with real-time usage data provides new insight into engagement with a reproductive health app used for menstrual tracking. Findings underscore the importance of PEOU for sustained engagement and point to gains in users’ confidence in their menstrual knowledge. Future work should include more diverse participants.

## 1 Introduction

The global mHealth sector has rapidly expanded in recent decades, driven in part by widespread smartphone adoption, increasing consumer interest in health tracking, and the accelerated development of digital health tools during the COVID-19 pandemic (1, 2). Menstrual cycle tracking apps are among the most widely downloaded health-related mobile applications and are used by millions of menstruating individuals worldwide (3), with implications for menstrual literacy, reproductive autonomy, and self-management of reproductive health. Recent scoping reviews show that users are motivated to engage with these apps for a variety of reasons - including menstrual education, contraception, and conception (4, 5). Additional motivations include gaining awareness of body functions, verifying menstrual regularity, preparing for upcoming menses, understanding reactions to different cycle phases, guiding conversations with healthcare providers, and identifying bleeding irregularities (3, 4, 6). Some studies suggest that menstrual cycle tracking apps may support menstrual literacy and empower users through improved body awareness (7–10), while others argue that such apps rely heavily on a user's pre-existing health literacy (11). Although the broader literature often refers to these tools as reproductive health apps, this study focused on menstrual cycle tracking functionality in a reproductive health app.

Despite their popularity and potential as health tools, many menstrual cycle tracking apps face major limitations in scientific validation, standardized language, and consistency in user experience (4, 12–14). High attrition rates are another well-documented challenge, with 71% of users disengaging from apps within 90 days (15). This is reflected in public health research more broadly, which also sees 75%–90% of participants drop out before study completion (16). Reported reasons for this disengagement include poor user design, technical issues, lack of trust, and perceived irrelevance or inaccuracy (4, 12, 17, 18).

The Technology Acceptance Model (TAM) has been used in mHealth research to explain adoption and engagement patterns, highlighting factors like perceived usefulness (PU) and perceived ease of use (PEOU) as central to predicting behaviour (19–22). However, despite its growth in other digital health areas, the model has been applied only sparingly to menstrual cycle tracking apps. Given the size of the user base and the rapid growth of menstrual app research, this gap warrants further attention.

In addition to the existing TAM models, another tool developed and validated through application in practice is the Mobile App Rating Scale (MARS) (23). Overall, the main dimensions of the MARS include some elements parallel to the TAM model such as assessments of app engagement, functionality, aesthetics, quality of information, impact on behaviour–all of which can be adapted to both app reviewers and users (23). To date, the MARS has been successfully applied in two recent studies assessing MC apps (12, 24). A key finding in both studies is that although MC apps have the potential to be helpful self-monitoring tools, many currently available lack evidence-based educational content, quality assessment measures, and inclusive design, thus potentially underserving their users (6, 12, 24, 25).

To our knowledge, no studies have integrated the TAM with granular, real-time engagement metrics in the context of menstrual cycle tracking apps. Building on this gap, we employed a 90-day app-based intervention using a reproductive health app with the functionality of menstrual cycle tracking to explore how patterns of engagement relate to menstrual literacy and TAM constructs and assess user-perceived app quality by employing the user-version of the Mobile App Rating Scale (uMARS). Our specific objectives were to 1) determine whether baseline menstrual literacy and TAM constructs predicted subsequent app engagement; 2) examine changes in menstrual literacy and TAM constructs over time by engagement level; and 3) summarize user experience and perceived app quality by employing the uMARS at follow-up.

## 2 Materials and methods

### 2.1 Study design and recruitment

The University of Calgary and a professional Marketing & Media Agency partnered to develop a social media ad campaign using Instagram and Facebook assets. The ads were set up to target 18-35-year-old menstruating individuals living in Canada. Respondents to the ads were sent to a landing page where they accessed an online survey which included the consent form and a questionnaire (Qualtrics XM; Qualtrics International Inc., USA). Approval for the study was received by The Conjoint Health Research Ethics Board (CHREB) at the University of Calgary (REB22-1025), and the study adhered to the guidelines established by the Declaration of Helsinki.

### 2.2 Participants and eligibility criteria

Eligible participants were menstruating individuals aged 18–35 years, assigned female sex at birth (i.e., hormones consistent with female biological sex), who reported menstrual cycle lengths between 21 and 35 days over the previous three months (26, 27) and had access to an iPhone. Participants completed an initial eligibility (first) survey, where they had the option to sign up to engage with a Female Health Menstrual Cycle Tracking App for free.

### 2.3 App-Based intervention and data collection

Instructions on how to download and use the app were sent to the email provided by the participant. Once the individuals were onboarded, they were assigned a study ID associated with their own app account. A follow-up (second survey) was sent to the participants’ email at the end of 90-day app intervention. The surveys were based on identified gaps in the literature and focused on participants’ demographics, menstrual literacy, tracking methods and the TAM constructs.

The intervention involved a proprietary female-health menstrual cycle tracking app provided by the company partner. Participants accessed the app as end users, and study procedures were limited to onboarding, app use, and collection of participant-reported and usage-based outcomes; the research team did not have access to the full backend configuration or developer-side settings. Participant-facing functions that were directly observable in the study data included menstrual cycle event logging, symptom entries, exercise tracking, and responses to in-app questions. Accordingly, app characteristics are described only to the extent they were observable within the participant experience.

### 2.4 Measures

App engagement for each participant was measured based on the frequency of interactions with the app's features, including participant updates, menstrual cycle (MC) event logging, exercise tracking, symptom entries, and responses to in-app questions. Each interaction generated a unique date/time stamp linked to the participant's study ID. For each participant, the total number of unique engagement timestamps over the 90-day study period was summed to create an overall engagement score to describe how “engaged” each participant was. Engagement ranged from 1 to 835 timestamps per participant.

Participants were then sorted by frequency of engagement, and the relative frequency or percent contribution of each participant's engagement to the total participant engagement dataset was calculated. Based on this distribution, participants were stratified into two groups (low-engagement and high-engagement) for subsequent analyses. These interactions reflected participant-entered actions captured through study-linked app usage data; the research team did not have access to backend-controlled features that were not directly observable in the participant data such as passive sensor data, anonymous chat functions, or others.

### 2.5 Menstrual literacy

Menstrual literacy outcomes included confidence in menstrual knowledge and perceived information sufficiency, both assessed using Likert-type items, with higher scores indicating greater confidence or perceived information sufficiency.

### 2.6 TAM constructs

Each TAM construct—perceived usefulness (PU), perceived ease of use (PEOU), and trust—was measured using Likert-scale items (three items for PU and trust; four for PEOU). Item scores within each construct were averaged to create composite scores, with higher values indicating greater perceived usefulness, ease of use, or trust.

### 2.7 Statistical analysis

All analyses were conducted using R version 2024.12.1 + 563 (28). Descriptive statistics were reported as means and standard deviations (SD) for continuous variables, and as counts and proportions for categorical variables. Group differences in demographic characteristics, contraceptive use, period tracking behaviours, and menstrual literacy between low- and high-engagement users were assessed using Welch's *t*-tests for continuous variables (e.g., age, age at menarche) and chi-square or Fisher's exact tests for categorical variables, as appropriate. Results are presented for the total sample and stratified by engagement group. For variables where participants could select more than one response, results were summarized using counts and percentages only. Follow-up survey responses to the uMARS were summarized using mean scores (23).

A multivariate logistic regression model was used to assess whether baseline menstrual literacy variables and TAM constructs were associated with app engagement status (high vs low), based on previously piloted methods (29). Participants with missing data on relevant key variables were excluded from the corresponding analysis. Odds ratios (OR) and 95% confidence intervals (CI) were reported for all predictors, and statistical significance was set at p < 0.05.

To assess changes in menstrual literacy over the 90-day app period, paired t-tests were conducted separately for high- and low-engagement groups among participants who completed both baseline and follow-up surveys. Outcomes included confidence in menstrual knowledge and perceived information sufficiency. Cohen's d was calculated to estimate within-subject effect sizes for change over time.

To explore whether TAM constructs changed over time and whether this differed by engagement level, linear mixed models (LMMs) were fitted for each construct (PU, PEOU, trust). Each model included fixed effects for timepoint (baseline vs follow-up), engagement group (low vs high), and their interaction, with random intercepts for participants to account for repeated measures. Estimated marginal means (EMMs) were used for *post hoc* comparisons. All tests were two-tailed, with statistical significance set at p < 0.05.

## 3 Results

**Figure 1:**
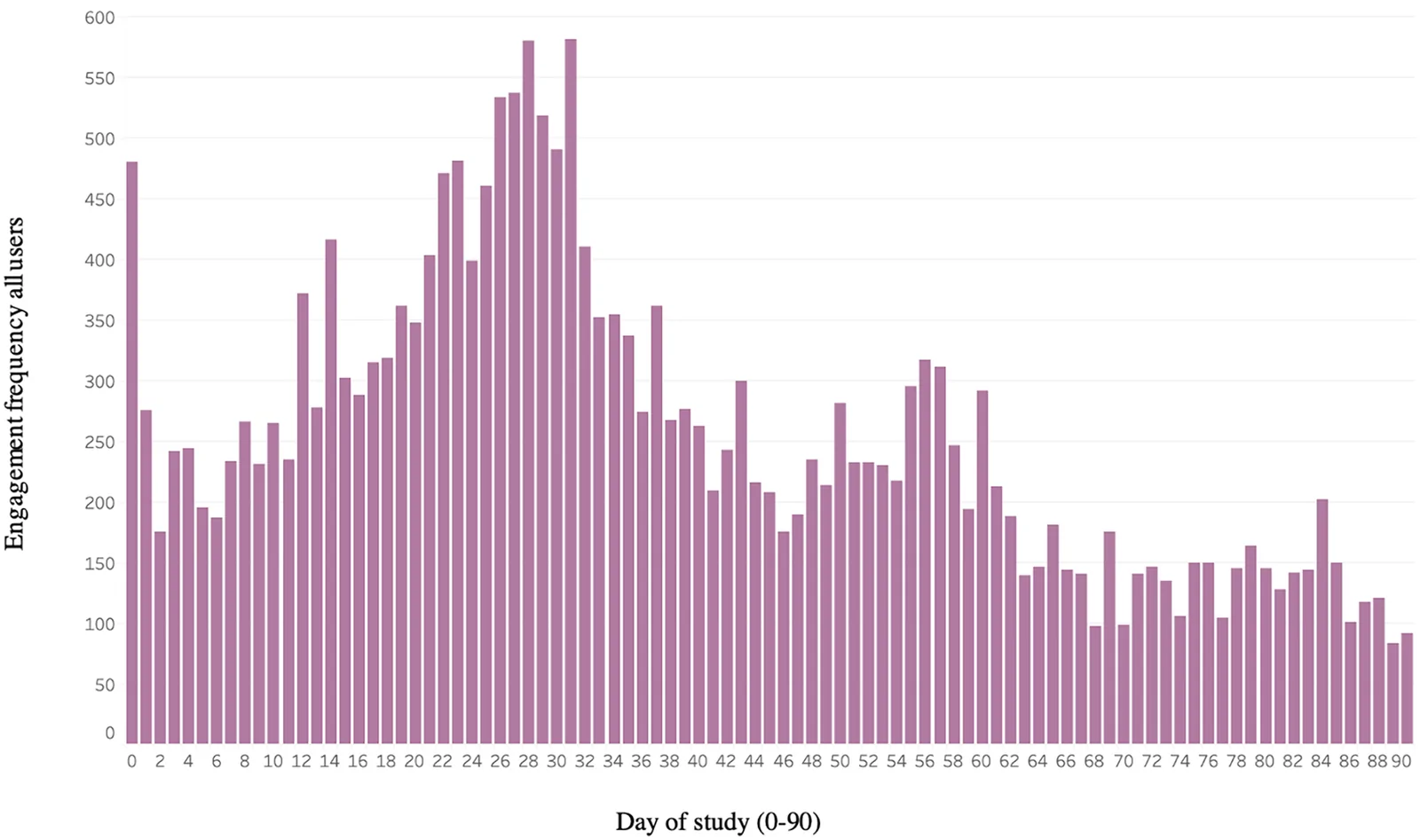
Engagement frequency distributions of all users (n = 293) by study day.

The study recruited 293 eligible participants who downloaded and used the app. The first participant enrolled on 16-Nov-22, the last on 31-Mar-23 with data collection completed on 01-Jul-23. Day 0 of the study was used as the study start date and day 90 was considered the end date for all participants (see Figure 1). A total of 127 participants (43%) completed the 90-day follow-up survey. The completion rates of the follow-up survey varied between engagement groups with 79% (33/42) of high engaged users completing compared to 37% (94/251) of low-engaged users.

### 3.1 Demographics

App users primarily identified as white/Caucasian (74%), female (98%) and had some university/bachelor's/master's or professional degree (78%). No significant demographic differences were found between low and high engaged users. The majority of participants indicated that they already keep a record of both when their period occurred (89%) and to predict future periods (88%). The most popular apps listed were Flo (26%) and Clue (16%). The most common reasons for tracking their period were to “be prepared” (69%) and “be aware of how their bodies were doing” (54%). When asked, “How confident do you feel about your knowledge of the menstrual cycle?” 73% of users indicated somewhat confident or not confident at all. Only 27% of users were confident or extremely confident. Additionally, 57% of users stated that they had some information or were unsure they had any information at all regarding their period and its symptoms (See Appendices A–F).

### 3.2 Engagement variable

Participants were sorted from highest to lowest engagement, and the relative frequency or percent contribution to the overall app engagement was calculated. The distribution of engagement was strongly right-skewed (median = 47, mean = 88.2, range = 1–835; skewness = 2.71), with a small number of highly active users accounting for a disproportionate share of activity. The top two users appeared 835 times and 669 times, respectively, accounting for approximately 6% of the total app engagement, and the bottom seven users, with one date/time stamp, contributed less than 1% to the overall engagement. The cumulative percentage of frequencies was calculated, and the point at which cumulative engagement reached 50% was identified, establishing two equal groups of app engagement frequencies (high and low engagement). The top engaged users (n = 42) contributed the same amount to the overall app engagement as the bottom engaged users (n = 251). Therefore, the low-engaged group included 86% (251/293) of all app users with an engagement frequency range of 1-183 times over the study duration. The high-engaged group included 14% (42/293) of app users, with an engagement frequency range of 187-835. This cumulative-contribution threshold was chosen deliberately over a median split of individual scores or fixed quantiles (e.g., tertiles or quartiles). Given the pronounced skew, such divisions would have grouped similar low-frequency users while obscuring the small, highly active subset that accounted for half of all engagement. The cumulative approach instead reflects this concentration of engagement in a small group of users.

**Figure 2:**
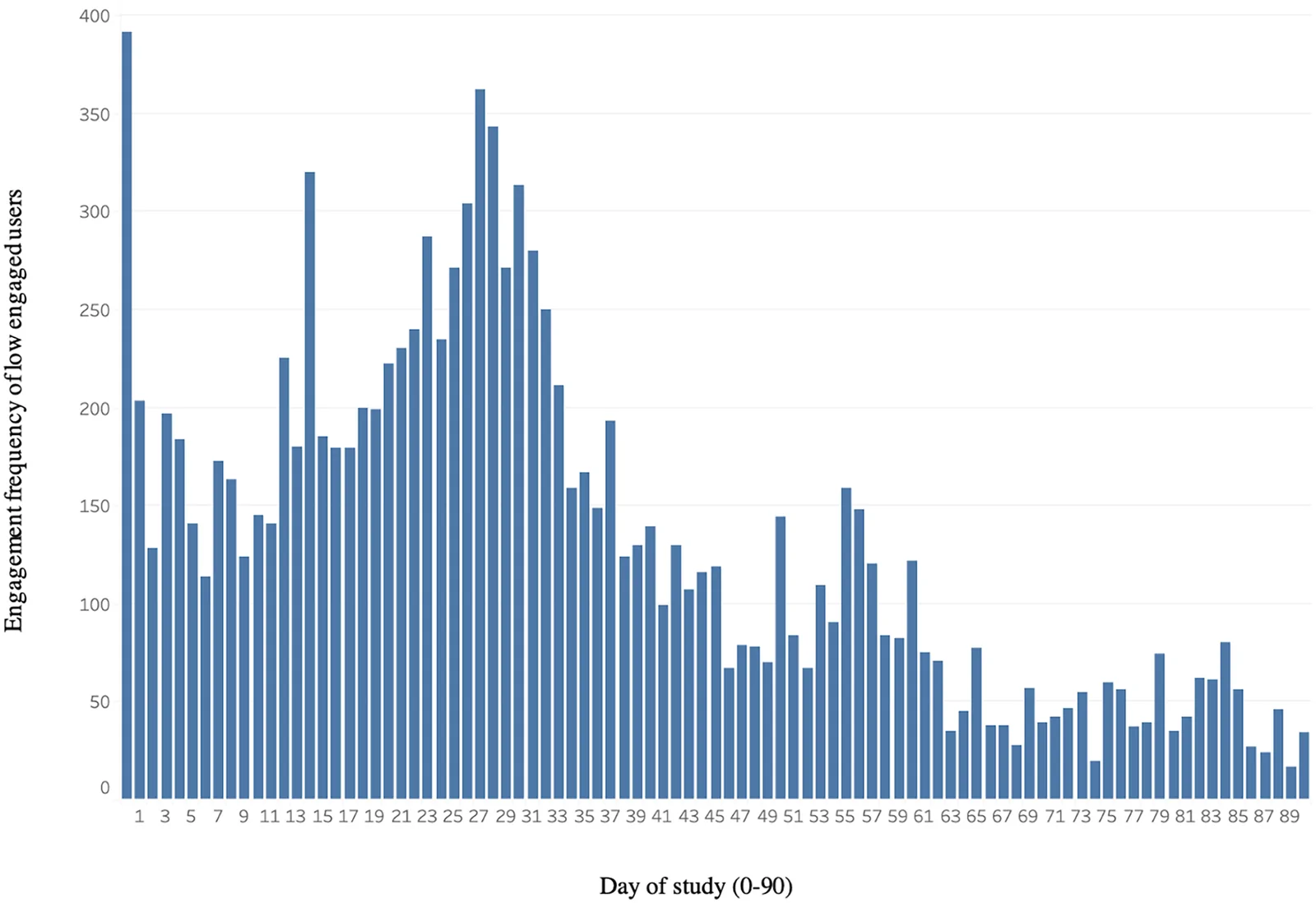
Engagement frequency distributions of the low app engagement group (n = 251) by study day.

**Figure 3:**
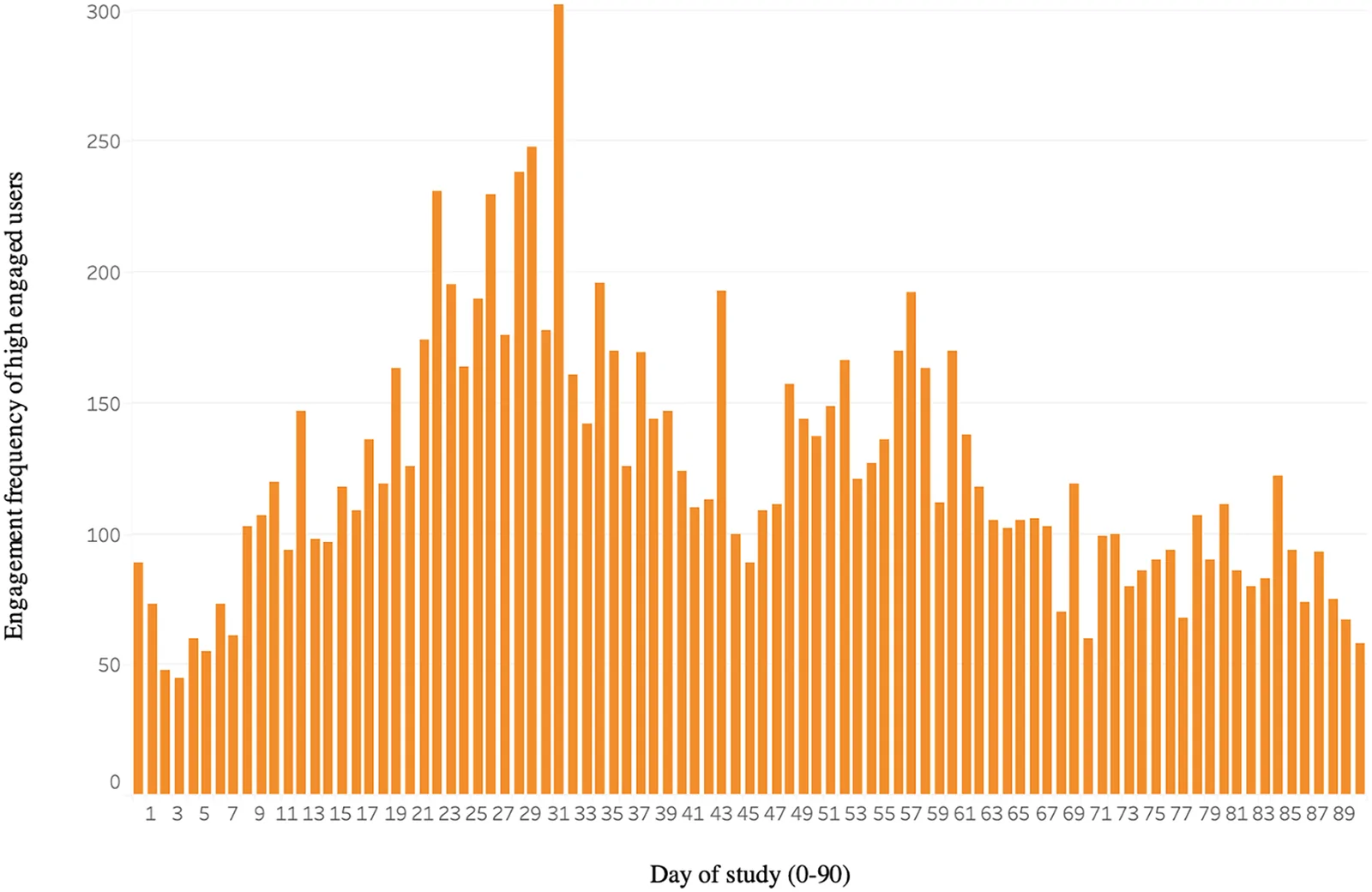
Engagement frequency distributions of the high app engagement group (n = 42) by day of study.

Figures 1–3 illustrate the daily distribution of engagement for all users stratified by group. In both low and high-engaged groups, peaks in engagement were observed between days 26-31 (see Figures 2, 3). A second peak was observed at ∼day 56 and a third at ∼day 84. In the lower engaged group, an additional large peak was observed on day 14. The lower engaged group registered the most app engagement on day 0 (sign up) and this followed with a drop off engagement at ∼day 34. The higher engaged users had a more consistent pattern of engagement up until the end of the study (day 90).

Table 1 summarizes engagement with key app features. Nearly all users logged menstrual cycle events (98%), while fewer used the symptom (73%) and exercise tracking features (42%). Within the users of each feature, the higher-engagement subgroup was largest for cycle event logging (28%), followed by symptom (11%) and exercise (4%) features, consistent with cycle tracking being the app's most-used feature.

**Table 1:** Frequency of app feature engagement by total participants, low and high engagers.

App Feature (n, %)	Total	Low	High
Cycle Event	286 (98)	205 (70)	81 (28)
Answer Date	269 (92)	233 (80)	36 (12)
Symptom Date	214 (73)	181 (62)	33 (11)
Exercise Date	123 (42)	111 (38)	12 (4)

n is the total number of users of each feature; % is relative to the total app user sample (N = 293). Users of each feature were divided into lower- and higher-engagement subgroups based on their interaction frequency with that specific feature, so the two subgroups sum to the feature total. These subgroups are exploratory and derived separately per feature to provide additional granularity on the distribution of engagement within each feature. They do not correspond to the study-level low- and high-engagement groups (n = 251 and n = 42) used in Tables 2–4. Therefore the subgroup split differs across features.

### 3.3 Baseline menstrual literacy and TAM

A multivariate logistic regression was conducted to explore whether baseline TAM constructs and menstrual literacy variables predicted engagement status (high vs. low). None of the predictors (PU, PEOU, Trust, confidence in knowledge, or information sufficiency) were statistically significant (all p > 0.05) (see Table 2).

**Table 2:** Exploration of TAM constructs and menstrual cycle literacy on app engagement group.

Predictor	Coefficient (*β*)	Std. Error	Odds Ratio (OR)	95% CI for OR	*p*-value
Perceived Usefulness (PU)	−0.214	0.294	0.81	0.46–1.45	0.465
Perceived Ease of Use (PEOU)	0.190	0.326	1.21	0.65–2.33	0.554
Trust	−0.103	0.180	0.90	0.63–1.28	0.568
Confidence	0.190	0.241	1.21	0.75–1.95	0.432
Information	−0.007	0.322	0.99	0.53–1.88	0.983

### 3.4 Menstrual literacy change

Paired *t*-tests were conducted separately for each engagement group to assess within-subject changes in menstrual literacy outcomes from baseline to follow-up (see Table 3). Among high-engagement users, mean confidence in menstrual knowledge significantly increased from pre- (M = 2.26, SD = 0.77) to post intervention (M = 2.68, SD = 0.70), *t*(30) = 2.83, *p* = 0.008, reflecting a medium effect size (Cohen's *d* = 0.51). However, there was no significant change in information sufficiency (*p* = 0.66).

**Table 3:** Mean changes in menstrual literacy scores from baseline (pre) to follow-Up (post) by engagement group.

Measure	Engagement Group	Pre Mean (SD)	Post Mean (SD)	*p*-value	Cohen's *d*
Confidence in Knowledge	High	2.26 (0.77)	2.68 (0.70)	0.008^a^	0.51
	Low	2.21 (0.80)	2.41 (0.78)	0.007^a^	0.25
Information Sufficiency	High	2.40 (0.50)	2.52 (0.68)	0.660	–
	Low	2.32 (0.62)	2.43 (0.71)	0.159	–

a Significant result.

Among low-engagement users, mean confidence in menstrual knowledge significantly increased from pre- (M = 2.21, SD = 0.80) to post-intervention (M = 2.41, SD = 0.78, *t*(89) = 2.75, *p* = 0.007) though with a smaller effect size (Cohen's *d* = 0.25) compared to high-engaged users. Information sufficiency again also showed no significant change (*p* = 0.159).

### 3.5 Pre/post change in TAM constructs

To assess whether TAM constructs changed over time and differed by engagement level, linear mixed models (LMMs) were run for each construct (Table 4). For PU, no significant effects were observed for time, group, or their interaction (all *p* > 0.05). For Trust, results were similarly non-significant.

**Table 4:** Estimated marginal means and linear mixed model effects for TAM constructs by engagement group.

	Engagement, mean score			
Construct	High n = 33 pre; post	Low n = 90 pre; post	Main Effect (Time)	Interaction (Group × Time)
PEOU	4.23; 4.23	4.18; 3.79	^a^b = −0.38, p < 0.001	^a^b = 0.39, p = 0.032
PU	3.98; 4.10	3.91; 3.76	b = −0.15, p = 0.098	b = 0.27, p = 0.122
Trust	2.72; 2.59	2.72; 2.63	b = −0.09, p = 0.306	b = −0.04, p = 0.800

PEOU, perceived ease of use; PU, perceived usefulness.

a Significant result.

PEOU, however, showed a significant time effect (b = −0.38, p < 0.001), indicating a decline from baseline to follow-up. A significant interaction between time and engagement (b = 0.39, *p* = 0.032) indicated that low-engaged users experienced a larger drop in PEOU over time, while high-engaged users remained stable.

Estimated marginal means (EMMs) supported this interaction. Among low-engaged users, PEOU declined significantly (4.18 to 3.79; *p* < 0.001), while high-engaged users remained stable (4.23 to 4.23; *p* = 1.00). At follow-up, high users reported significantly higher PEOU than low users (*p* = 0.022).

### 3.6 uMARS

Among the 127 participants who completed the follow-up, uMARS scores indicated moderate perceptions of app quality. Mean ratings were highest for information quality (M = 3.6) and lowest for engagement (M = 2.6). The overall app quality mean score was 3.1, with an average star rating of 3.0. The subjective item “Would you pay for this app?” received the lowest rating (M = 1.0) (see Table 5).

**Table 5:** Follow-up questionnaire – uMARS.

App Quality uMARS items	Total n = 127
Mean score (out of 5)	
**Engagement** (fun, interesting, customisable, interactive, has prompts (e.g., sends alerts, messages, reminders, feedback, enables sharing)	2.6
**Functionality** (app functioning, easy to learn, navigation, flow logic, and gestural design of app)	3.2
**Aesthetics** (graphic design, overall visual appeal, colour scheme, and stylistic consistency)	3.0
**Information** (Contains high quality information (e.g., text, feedback, measures, references) from a credible source)	3.6
**App Quality Mean Score (out of 5)**	**3**.**1**
**Subjective quality**	
Would you recommend this app to people who might benefit from it? (1 = not at all; 3 = maybe; 5 = definitely)	2.1
Would you pay for this app? (1 = definitely not; 5 = definitely yes)	1.0
What is your overall (star) rating of the app? (1 = one of the worst apps I've used; 3 = average; 5 = one of the best apps I've used)	3.0

## 4 Discussion

This study examined engagement with a reproductive health app that tracks menstrual cycles over a 90-day period, using real-time usage data linked with TAM constructs, menstrual literacy indicators, and user-perceived app quality. We compared high- and low-engagement users to explore patterns of app use, changes in menstrual literacy and TAM constructs over time, and perceptions of app quality captured by the uMARS. Taken together, these findings add to the growing literature on menstrual cycle tracking apps by highlighting the importance of perceived ease of use for sustaining engagement and the potential for menstrual cycle tracking apps to support confidence in menstrual knowledge. However, the study sample was relatively homogeneous, which limits generalizability to more diverse populations. Engagement patterns was also uneven across participants, with a small subgroup accounting for a disproportionate share of app use and follow-up completion.

### 4.1 Engagement and retention in a reproductive health app

In line with this focus on engagement patterns, our study showed that highly engaged users demonstrated greater app use across all measures: they interacted with the app more frequently over the 90-day period and were substantially more likely to complete the follow-up survey. While only 14% (42/293) of participants were categorized as highly engaged, this group contributed half of all recorded app interactions, equivalent to the combined engagement of the remaining 86% (251/293). Most participants (86%) fell into the low-engagement category, with a 90-day follow-up survey completion rate of 37% (94/251), compared to 79% (33/42) among high-engagement users. These completion rates are consistent with expectations, given participant attrition is a well-documented challenge in mHealth research and app-based interventions (10, 15, 16, 18, 30–40). Reported attrition rates and definitions of engagement and retention vary widely depending on the app's type and purpose (18) but it is estimated that one in four apps are used only once after download (41), and 71% of users disengage within 90 days—figures comparable to public health research, where 75%–90% of participants typically disengage over the course of a study (15). App-based interventions for chronic disease have reported attrition rates as high as 98%, with most users engaging briefly before dropping out (37). In their review, Boucher and Raiker (30) reported that adherence ranged widely, from 3 to 95% with an average of 45%; minimal usage ranged from 21 to 88%, while sustained usage, defined as beyond six weeks, ranged from just 0.5 to 29%. These estimates align with the 25% sustained usage rate reported by Coughlin et al. (32) and with evidence that longer interventions face additional challenges, with median retention across app-based studies reported as approximately 5.5 days (32, 38).

In the context of these widespread challenges with app-based attrition and relative to existing research, our 90-day follow-up completion rate of 79% (33/42) for the high engagement group is particularly positive, even though it reflects a smaller sample (33/293) of the total sample. Meanwhile, the low-engagement group had a 90-day follow-up completion rate of 37% (94/251). Despite being the lower-engagement group, this retention and follow-up completion still exceed the overall averages reported in the literature, suggesting that participants in this study were generally more motivated to remain engaged and complete the study (10, 15, 18, 30–40).

We speculate that several factors may have contributed to the overall study completion and above-average retention observed in our sample. In a recent scoping review conducted by our team, we found that menstrual tracking app studies often recruit large participant numbers, with growing user interest driven by motivations such as menstrual cycle tracking, contraception and conception planning, and educational purposes (4). Although we did not specifically ask participants about their motivation to participate, prior work suggests interest in reproductive health research is often rooted in altruism and the perception that it addresses important and under-researched areas (42). Moreover, higher retention may be influenced by having a clinical condition relevant to the app or belonging to a niche, highly motivated population with a strong personal drive to use the app—both of which may apply to reproductive health tracking (18).

### 4.2 Menstrual cycle-linked engagement patterns

Daily engagement patterns showed distinct peaks in app use that appeared to align with typical menstrual cycle timing. Across all users, engagement peaked between days 26–31, with secondary peaks around days 56 and 84, corresponding to approximately 30-day intervals. Based on a large-scale analysis of 124,648 app users (26), the average menstrual cycle length is approximately 29.3 days, which aligns with the 30-day spacing between engagement peaks in our study. We therefore infer that many participants were more likely to both sign up and subsequently engage with the app at or near the first day of their cycles, coinciding with the onset of menstruation, even though recruitment was on a rolling basis and independent of cycle timing. The more pronounced spike on day 14 in the low-engagement group is less easily explained but may reflect ovulation-related tracking among some users or an initial period of exploratory use followed by disengagement, consistent with broader mHealth research showing that longer interventions are associated with higher attrition (38).

We make these inferences cautiously and acknowledge the limitations of assuming uniformity in menstrual cycle phases and duration. As highlighted by Elliott-Sale et al. (43), such assumptions are challenged by issues of validity, reliability, and causality, and the commonly cited 28-day cycle applies to as few as 10% of menstrual cycles (44). Given that we are describing general engagement trends, rather than making individualized lifestyle or training recommendations, we consider these assumptions appropriate within the scope of the current study.

Feature-level engagement further supports these patterns. In our sample, 98% of participants used the menstrual cycle events feature at least once, 73% engaged with symptom tracking, and 42% used the exercise tracking feature. The near-universal use of cycle tracking suggests it was the primary driver of app engagement, consistent with usage spikes that likely coincided with menstrual cycle events. By contrast, lower uptake of symptom and exercise tracking may indicate that these features were perceived as less valuable, less usable, or more burdensome to incorporate into daily routines. One likely explanation is that the study's recruitment materials specifically highlighted menstrual cycle tracking, which may have shaped participant expectations and focused their engagement on that feature—a pattern echoed in baseline survey responses, where most participants reported already tracking their periods (89%) and regularly predicting future cycles (88%).

Several external studies report usage patterns consistent with our findings. A 2024 survey of period-app users aged 14–54 found that 86% reported entering more data while on their period, with many users primarily logging information during menstruation rather than daily; only about one-third reported inputting data every day, and 38% indicated that tracking was “not a priority” (10). Similarly, Rampazzo et al. (45) found that menstrual cycle tracking was the most common reason for using these apps (61%), followed by achieving pregnancy (22%), a sense of community (9%), and avoiding pregnancy (8%). Hong et al. (33) reported that 62% of Gen Z and millennial users use period-tracking apps primarily to predict their next menstrual cycle. The physical symptoms and inconveniences associated with menstruation may therefore motivate users to track for better self-management, as reflected in our participants’ reasons for tracking: “to be prepared” (69%) and “to be aware of how my body is doing” (54%). Studies have also shown increased engagement among users trying to conceive (10, 45, 46) and a 2022 survey found that 30% of respondents considered ovulation prediction the most valuable aspect of their period app (47). This latter reason appears less relevant in our sample, as only 6% of participants reported using the app for conception-related tracking, despite the peak in engagement on day 14.

### 4.3 Changes in menstrual literacy and implications for menstrual health

Over the 90-day intervention, confidence in menstrual knowledge increased in both high- and low-engagement groups, with a larger effect observed among highly engaged users. In contrast, perceived information sufficiency did not change significantly for either group. Notably, baseline confidence and information sufficiency did not predict engagement status in this sample, despite earlier work from our group showing that users with higher confidence in menstrual knowledge were less likely to use menstrual tracking apps relative to non-users.

Our regression analysis revealed that neither of the menstrual literacy constructs predicted engagement status, nor were there any baseline differences between high- and low-engaged users. This contrasts with findings from a recent study by our team, which used a similar regression approach and observed a negative correlation between confidence in knowledge and app use. Additionally, in that earlier study, information sufficiency was significantly higher among app users compared to non-users (29). The discrepancy between our two studies suggests that the confidence gap between high- and low-engagement users may be less pronounced than the gap observed between app users and non-users.

The increase in confidence over time, even among low-engagement users, suggests that menstrual tracking apps may support users’ confidence in their menstrual knowledge even at relatively low levels of use. Because perceived information sufficiency did not change, these findings point to gains in confidence rather than demonstrable increases in knowledge itself; they nonetheless support the potential of such apps as tools for enhancing menstrual literacy (4, 48–50), particularly for individuals who begin with lower confidence or limited information. However, the lack of change in perceived information sufficiency points to a possible disconnect where users may feel more confident, but do not necessarily perceive that they have access to all the information they need. This discrepancy raises questions about how menstrual literacy is defined and measured, and whether current instruments capture the full range of knowledge, understanding, and self-advocacy skills relevant to menstrual and reproductive health.

These findings highlight several areas for future research. First, it will be important to clarify which aspects of app content and functionality contribute most strongly to gains in confidence and whether these effects persist beyond the active intervention period. Second, more nuanced measures of menstrual literacy may be needed to differentiate between perceived confidence, objective knowledge, and the ability to use information in clinical or everyday decision-making. Finally, understanding whether literacy gains plateau over time, and whether this plateau contributes to disengagement, could inform the timing and design of in-app educational content.

### 4.4 TAM constructs, perceived ease of use and app quality

Our multivariate logistic regression analysis showed no significant baseline differences in the TAM constructs—perceived usefulness (PU), perceived ease of use (PEOU), and trust—between high- and low-engagement users. However, follow-up analyses showed that PEOU declined significantly over time among low-engagement users, whereas high-engagement users reported stable PEOU and significantly higher scores at follow-up compared with low-engagement users. These findings suggest that PEOU may be an important factor in shaping user engagement and retention, particularly among users who interact with the app less frequently. It is important to reiterate that these results are based on user experiences with a single app used in this intervention and may not be fully generalizable to other menstrual tracking applications.

The decline in PEOU among low-engagement users may be shaped by several factors. In our study, PEOU was assessed through items related to learning how to use the app, interacting with it in a clear and intuitive way, and becoming proficient in its use (21, 22, 51). Some participants may have encountered frustration, unmet expectations, or found the app less intuitive or too tedious to use over time. Notably, 88% of our sample reported regularly tracking and predicting their cycles, often using other apps. It is possible that some users returned to those familiar platforms, which they may have found easier to navigate, contributing to drop-off in use of the study app. In contrast, high-engagement users may have formed positive early impressions of the app or entered the study with higher levels of digital literacy, resulting in stable PEOU over time. As a TAM construct, PEOU appears to be closely tied to sustained engagement and highlights the importance of user-friendly app design.

It is important to note, however, that due to our study design, we cannot determine the direction of this relationship. Because baseline PEOU did not predict subsequent engagement status, the decline in PEOU among low-engaged users may reflect reduced engagement rather than cause it. Users who disengaged early may have subsequently perceived the app as less easy to use possibly due to less exposure to and less chance to familiarize themselves with it. It is also possible that this relationship is reciprocal, with declining ease of use and declining engagement influencing one another over the 90-day period. Identifying these factors would require more frequent measures throughout the study period, something which future studies may consider.

High attrition rates are common across mobile health apps, and our findings align with broader literature on app use and retention (18). Lin et al. (52) reported that average time users spend on an app can be as brief as 9.3 s, emphasizing the critical role of ease of use in shaping the initial user experience. This factor is equally important for long-term engagement, as technical issues or the perception that an app is too difficult to use can often lead to early dropout and lower retention rates (18, 31). Users consistently report valuing “participant-centric design” (40), ease of use, and an intuitive interface as key features that enhance their user experience (10, 35).

Challenges related to PEOU also intersect with broader issues that may contribute to app drop-off. These include concerns about privacy, frustration with inaccurate cycle predictions, and the stress such inaccuracies may cause. The lack of regulation in the menstrual tracking app market further exacerbates these issues, resulting in widely variable app quality (4, 10). In our study however, overall app quality, measured using the uMARS, had a mean score of 3.1, with a corresponding average user star rating of 3.0. These findings are in line with a recent review by Ko and colleagues (53), which reported a mean app quality score of 3.33 across 34 menstrual tracking apps.

## 5 Strengths and limitations

A major strength of our study is the integration of real-time engagement data from a female reproductive health app tracking menstrual cycles over a 90-day period with pre- and post-intervention survey data that included constructs from the TAM. To our knowledge, this is the first study to apply the TAM alongside direct app usage data in the context of menstrual tracking. This approach builds on our previous cross-sectional research comparing survey responses between app users and non-users (29), offering a more nuanced, behaviour-based understanding of engagement.

Access to actual app usage data allowed us to pilot a novel engagement classification system, categorizing participants into high and low engagement groups based on real user behaviour rather than relying solely on self-report. This enabled more meaningful group comparisons, particularly regarding differences in TAM constructs, and addresses a common challenge in digital health research, where engagement is often inconsistently defined and typically measured through self-reported app use data (30, 54, 55). Our operationalization of engagement may provide a useful model for future studies exploring app-based interventions, that seek to integrate usage and survey based-measures.

Several limitations should be noted. Although our social media recruitment strategy resulted in a large sample size (n = 293) within our target demographic, the participant pool was predominately White, highly educated, and female. This is unlikely to be representative of the broader Canadian population and may be particularly relevant to our menstrual literacy findings, as more educated individuals may already have greater baseline access to and familiarity with health information. More broadly, our team's scoping review of reproductive health apps found that a lack of diversity is one of the most pervasive issues in this literature, with study samples repeatedly characterized as Western, educated, and predominantly White (4). Our sample reflects this same pattern, limiting generalizability to more diverse populations (56–58).

Our recruitment was also limited by the use of research advertising channels, which likely reinforced the educational and demographic homogeneity of the sample. In addition, because the study app was available only on iOS, participation was limited to iPhone users, which may have introduced a possible socioeconomic bias.

A related limitation is that most participants reported prior use of menstrual tracking apps at baseline. Despite broad promotion, the study likely attracted a self-selecting group of tech-literate and already-engaged users, introducing potential selection bias and limiting the generalizability to individuals with lower digital literacy or no prior app experience. While the low-engaged group still showed moderate study retention, this may reflect a highly motivated subgroup, rather than a representative cross-section of app users (18).

A further limitation is the absence of qualitative data. Because our study was designed around the TAM, we did not directly ask participants about their perceptions, motivations, or reasons for disengagement, which limits our ability to explain why the observed engagement patterns occurred. Future research would benefit from mixed-methods designs that pair usage and survey data with qualitative inquiry, as demonstrated by Levy et al. (7), to capture the experiential and motivational factors underlying app engagement.

In addition, we could not independently characterize all app features that may influence engagement, such as notification architecture, customization options, or other backend design elements, because the app was provided through a proprietary agreement with the developer.

Finally, we were unable to recruit enough app non-users to explore how outcomes might differ for individuals with no prior exposure to menstrual tracking apps. Consequently, we could not fully assess how first-time users engage with and respond to app-based interventions over time—an area that warrants further investigation in future reproductive health research.

## 6 Future directions

These findings highlight several priorities for future research. First, studies should identify which specific aspects of app content and functionality contribute most strongly to gains in menstrual confidence and whether these effects persist beyond the active intervention period. Second, more nuanced measures of menstrual literacy are needed to distinguish between perceived confidence, objective knowledge, and the ability to apply information in clinical encounters and everyday decision-making. Future studies should incorporate validated menstrual or reproductive health knowledge assessments, such as questionnaires recently developed by Roux et al. (59) and Fischer et al. (60), alongside confidence measures to determine whether app use improves objective knowledge. Third, longitudinal work should examine whether literacy gains plateau over time and whether such plateaus contribute to disengagement, thereby informing the timing and design of in-app educational content. Finally, future research should use more inclusive recruitment strategies to better evaluate how these patterns generalize across racial, socioeconomic, and gender-diverse populations.

## 7 Conclusion

This study applied the TAM in combination with real-time engagement data from a female reproductive health app tracking menstrual cycles to explore user behaviour over a 90-day period. Although no baseline differences in TAM constructs were observed between high- and low-engagement users, the significant decline in perceived ease of use among low-engagement users over time suggests that PEOU may be a key factor in retaining user engagement. Additionally, both groups showed increased confidence in menstrual knowledge over time, indicating that even relatively low levels of app use may offer educational benefits related to menstrual literacy by supporting their confidence in their menstrual knowledge.

To our knowledge, this is the first study to integrate TAM constructs with real-time usage data in the context of menstrual tracking, offering a novel approach within the digital health and reproductive health space. At the same time, our findings underscore persistent challenges, including limited participant diversity and the self-selection of already-engaged, tech-literate app users. Taken together, our results contribute to the growing body of literature on menstrual health apps and provide a foundation for future research aimed at improving app design, user retention, and inclusivity in reproductive health technologies.

## Data Availability

The datasets presented in this article are not readily available because the data are not openly available due to participant confidentiality and sensitivity of reproductive health information. Requests to access the datasets should be directed to pdoyleba@ucalgary.ca.
