# Supplementary material for: Exploring engagement patterns in a reproductive health app using the Technology Acceptance Model
Source: Front Reprod Health. 2026 Aug 17;8:1904798. doi: 10.3389/frph.2026.1904798 (PMC13526922; doi:10.3389/frph.2026.1904798)
Supplement: Supplementary file 1 [file Supplementaryfile1.docx]

**Supplementary Materials**

**Appendix A**. Baseline Participant Demographics and Contraceptive Use Stratified by App Engagement Level

| Characteristics | Total population  N=293 | Low Engagement  n=251; 85.7% | High Engagement  n=42; 14.3% | *P* value |
| --- | --- | --- | --- | --- |
|  | Mean; ± SD | | |  |
| Age | 26; 5 (n=284) | 26; 5 (n=243) | 26; 5  (n=41) | 0.337^a^ |
| Age at onset of menarche | 12.4; 1.8  (n=286) | 12.3; 1.7  (n=244) | 12.4; 1.8  (n=12.4) | 0.570^a^ |
|  | n (%) | | |  |
| Gender identity  Female  Diverse identities (i.e. gender fluid, non- binary) | 287 (98)  6 (2) | 246 (98)  5 (2) | 41 (98)  1 (2) | 1^c^ |
| Racial or ethnic group  Caucasian (White)  BIPOC | 217 (74)  76 (26) | 181 (72)  70 (28) | 36 (86)  6 (14) | 0.491^b^ |
| Highest level of education  Some high school/high school  Some university/bachelor's/master's or professional degree  Trade certificate/Diploma  Some or completed college/CEGEP | 24 (8)  229 (78)  13 (4)  27 (9) | 21 (8)  194 (77)  10 (4)  26 (10) | 3 (7)  35 (83)  3 (7)  1 (2) | 0.254^c^ |
| Currently using an oral contraceptive  Yes  No  Not sure | 92 (31)  199 (68)  2 (1) | 81 (32)  169 (67)  1 (0) | 11 (26)  30 (71)  1 (2) | 0.243^c^ |
| Reasons for contraceptive use (select all that apply)  Contraceptive reasons  Making my cycle more regular  Reducing my period pain  Acne/skin problems  Reducing how heavy my bleeding is  So, I can skip or shift when I have my period  Other (e.g., endometriosis, PCOS, amenorrhea, fibroids, IUD, hormone regulation) | 71 (24) 31 (11) 47 (16) 21 (7) 47 (16) 9 (3)  6 (2) | 62 (25) 26 (10) 36 (14) 19 (8) 28 (11) 8 (3)  6 (2) | 9 (21) 5 (12) 5 (12) 2 (5) 4 (10) 1 (2) | - |
| Contraceptive advice source (select all that apply)  Healthcare practitioner (including: doctor, nurse, etc.)  Family  Friends  School  Advice from internet  Other | 76 (26)  15 (5)  18 (6)  1 (0)  8 (3)  4 (1) | 67 (27)  10 (4) 15 (6) 1 (0) 8 (3) 4 (2) | 9 (21)  5 (12) 3 (7)  0 (0) 42 (100) | - |

All participants were assigned female sex at birth. Diverse gender identities include: Two-spirit, non-binary, gender fluid, gender queer, questioning, gender non-conforming, demigirl, agender, transmasculine. Ethnic information may sum to more than 100% if respondents chose more than one category. This variable was dichotomized into two groups (White and BIPOC) to assess differences between app users and non-users.

a: Welch’s t-test

b: Chi-square

c: Fisher exact test

*: Significant result

**Appendix B**. Baseline Period Tracking Behaviours by App Engagement Level

| Characteristics | Total population  n=293 | Low  n=251 | High  n=42 | *P* value |
| --- | --- | --- | --- | --- |
|  | n (%) | | |  |
| Record when period has occurred  Yes  No | 259 (88) 29 (10) | 224 (89) 25 (10) | 35 (83) 4 (10) | 0.919 |
| Predict when next period will be  Yes  No | 259 (88) 32 (11) | 220 (88) 29 (12) | 39 (93) 3 (7) | 0.650 |
| Reasons for tracking period (select all that apply)  Be prepared  To conceive  Be aware of how my body is doing  Understand bodily’s reactions to different phases of my cycle  Inform conversations with healthcare providers  Other | 203 (69)  18 (6)  158 (54)  124 (42)  85 (29)  16 (6) | 175 (70)  16 (6)  134 (53)  107 (43)  69 (28)  15 (6) | 28 (67)  2 (5)  24 (57)  17 (41)  16 (38)  1 (2) | - |

**Appendix C**. Baseline Menstrual Cycle Literacy, Confidence, and Knowledge Sources by App Engagement Level

| Characteristics | Total population  n=293 | Low  n=251 | High  n=42 | *P* value |
| --- | --- | --- | --- | --- |
|  | n (%) | | |  |
| Perceive if period is normal  Yes – I’m definitely sure its normal  Yes – I think it might be normal  Unsure – don’t know either way  No – I think there might be something abnormal  No – I am certain there is something abnormal happening | 91 (31) 136 (46) 18 (6) 29 (10) 19 (7) | 79 (32) 109 (43) 18 (7) 28 (11) 17 (7) | 12 (29) 27 (64) 0 (0) 1 (2) 2 (5) | *0.047 |
| How did you decide if your period was normal?  Asked mother  Asked sister  Asked other female relatives  Asked friends  Learned in school  Asked doctor  Online search  Other | 29 (10) 3 (1) 2 (1) 21 (7) 63 (22) 77 (26) 74 (25) 23 (8) | 26 (10) 3 (1) 2 (1) 18 (7) 55 (22) 68 (27) 61 (24) 45 (18) | 3 (7)   3 (7) 8 (19) 9 (21) 13 (31) 9 (21) | 0.723 |
| What sources are trustworthy? (select all that apply)  Internet  Family  Friends  School programs  School teachers/university lecturers  Magazines  Health apps on mobile devices  Health professionals (doctors, nurses, gynaecologist)  Social media (e.g. Facebook)  Independent health organisations  Television shows  Blogs | 185 (63) 96 (33) 94 (32) 73 (25) 94 (32) 5 (2) 129 (44) 272 (93) 12 (4) 281 (96) 2 (1) 15 (5) | 158 (63) 84 (34) 81 (32)  62 (25) 74 (30) 4 (2) 109 (43) 233 (93) 11 (4) 241 (96) 1 (0) 13 (5) | 27 (64) 12 (29) 13 (31) 11 (26) 20 (48) 1 (2) 20 (48) 39 (93) 1 (2) 8 (19) 1 (2) 2 (5) | - |
| What have you been taught about MC (select all that apply)  Biology of the menstrual cycle  What is a regular menstrual cycle and symptoms  What is considered irregular cycles and symptoms  What to do if you think your cycle is irregular  How to manage symptoms  Scenarios and examples of managing your menstrual cycle  Menstrual products  Benefits of exercise for the menstrual cycle & symptoms  None  Other | 235 (80) 261 (89) 183 (63) 127 (43) 210 (72) 152 (52) 259 (88) 173 (59) 1 (0) 4 (1) | 198 (79)  206 (82)  156 (62)  107 (43)  179 (71)  130 (52)  247 (98)  143 (57)  1 (0)  3 (1) | 37 (88) 36 (86) 27 (64) 20 (48) 42 (100) 22 (52) 42 (100) 30 (71)  1 (2) | - |
| Confidence in knowledge about the menstrual cycle and its effect on health (e.g. bone health, mental health)  Not confident at all  Somewhat confident  Confident  Extremely confident | 46 (16)  167 (57)  61 (21)  18 (6) | 40 (16) 146 (58) 48 (19) 16 (6) | 6 (14)  21 (50)  13 (31)  2 (5) | 0.441 |
| Have sufficient information about your period and its symptoms  I have sufficient information about my period and its symptoms  I have some information but am unsure about a number of aspects  I am unsure if I have enough information at all | 125 (43)  153 (52)  15 (5) | 108 (43)  128 (51)  15 (6) | 17 (40)  25 (60)  0 (0) | 0.246 |

How did you decide if your period was normal? “Asked Father” was an option, but no participants chose this (0).

*: Significant result

**Appendix D.** Baseline Participant Demographics and Contraceptive Use Stratified by Follow-Up Completion Status

| Characteristics | Total population  N=293 | Completed FU  n=127; 43.3% | Lost to FU  n=166; 56.7% | *P* value |
| --- | --- | --- | --- | --- |
|  | Mean; ± SD | | |  |
| Age | 26; 5 (n=284) | 27; 5 (n=123) | 26; 5  (n=161) | 0.228^a^ |
| Age at onset of menarche | 12.4; 1.8  (n=286) | 12.3; 1.5  (n=125) | 12.5; 2.0  (n=161) | 0.256^a^ |
|  | n (%) | | |  |
| Gender identity  Female  Diverse identities (i.e. gender fluid, non- binary) | 287 (98)  6 (2) | 126 (99)  1 (1) | 161 (97)  5 (3) | 0.239^c^ |
| Racial or ethnic group  Caucasian (White)  BIPOC | 217 (74)  76 (26) | 91 (72)  36 (28) | 126 (76)  40 (24) | 0.491^b^ |
| Highest level of education  Some high school/high school  Some university/bachelor's/master's or professional degree  Trade certificate/Diploma  Some or completed college/CEGEP | 24 (8)  229 (78)  13 (4)  27 (9) | 9 (7)  102 (80)  4 (3)  12 (9) | 15 (9)  127 (77)  9 (5)  15 (9) | 0.725^c^ |
| Currently using an oral contraceptive  Yes  No  Not sure | 92 (31)  199 (68)  2 (1) | 37 (29)  88 (69)  2 (2) | 55 (33)  111 (67)  0 (0) | 0.277^c^ |

Note: Follow-Up, FU. All participants were assigned female sex at birth. Diverse gender identities include: Two-spirit, non-binary, gender fluid, gender queer, questioning, gender non-conforming, demigirl, agender, transmasculine. Ethnic information may sum to more than 100% if respondents chose more than one category. This variable was dichotomized into two groups (White and BIPOC).

a: Welch’s t-test

b: Chi-square

c: Fisher exact test

**Appendix E**. Baseline Menstrual Cycle Literacy by Follow-Up Completion Status

| Characteristics | Total population  n=293 | Completed FU  n=127 | Lost to FU  n=166 | *P* value |
| --- | --- | --- | --- | --- |
|  | n (%) | | |  |
| Perceive if period is normal  Yes – I’m definitely sure its normal  Yes – I think it might be normal  Unsure – don’t know either way  No – I think there might be something abnormal  No – I am certain there is something abnormal happening | 91 (31) 136 (46) 18 (6) 29 (10) 19 (6) | 42 (33) 60 (47) 5 (4) 12 (9) 8 (6) | 49 (30) 76 (46) 13 (8) 17 (10) 11 (7) | 0.727 |
| Confidence in knowledge about the menstrual cycle and its effect on health (e.g. bone health, mental health)  Not confident at all  Somewhat confident  Confident  Extremely confident | 46 (16)  167 (57)  61 (21)  18 (6) | 20 (16) 69 (54) 30 (24) 8 (6) | 26 (16)  98 (59)  31 (19)  10 (6) | 0.767 |
| Have sufficient information about your period and its symptoms  I have sufficient information about my period and its symptoms  I have some information but am unsure about a number of aspects  I am unsure if I have enough information at all | 125 (43)  153 (52)  15 (5) | 54 (43)  66 (52)  7 (6) | 71 (43)  87 (52)  8 (5) | 0.954 |

Note: Follow-Up, FU. *Fisher exact test

**Appendix F**. Baseline TAM Constructs by Follow-Up Completion Status

| Characteristics | Total population  n=293 | Completed FU  n=127 | Lost to FU  n=166 | *P* value |
| --- | --- | --- | --- | --- |
|  | Mean; ± SD | | |  |
| Perceived Usefulness (PU) | 3.86; 0.69 | 3.91; 0.72 | 3.82; 0.67 | 0.254 |
| Perceived Ease of Use (PEOU) | 4.10; 0.66 | 4.19; 0.63 | 4.04; 0.68 | 0.062 |
| Trust | 2.71; 0.97 | 2.73; 0.91 | 2.69; 1.03 | 0.724 |

Note: Follow-Up, FU. *Welch’s t-test
